# An updated review on abnormal epigenetic modifications in the pathogenesis of systemic lupus erythematosus

**DOI:** 10.3389/fimmu.2024.1501783

**Published:** 2025-01-06

**Authors:** Xingyu Zhou, Shengnan Zhou, Yaping Li

**Affiliations:** Department of Dermatology, Hunan Key Laboratory of Medical Epigenomics, Second Xiangya Hospital, Central South University, Changsha, China

**Keywords:** epigenetics, systemic lupus erythematosus, DNA methylation, histone modification, noncoding RNAs, RNA methylation, biomarkers

## Abstract

Systemic lupus erythematosus (SLE) is a chronic autoimmune disease. The inconsistent prevalence of SLE between monozygotic twins suggests that environmental factors affect the occurrence of this disease. Abnormal epigenetic regulation is strongly associated with the pathogenesis of SLE. Epigenetic mechanisms may be involved in the development of lupus through DNA methylation, histone modification, noncoding RNAs, and other modifications. This review aims to show numerous studies as a treasure map to better understand the effects of aberrant epigenetic modification in the onset and development of SLE, which will benefit the current basic research and provide potential diagnostic biomarkers or therapeutic targets for SLE.

## 1 Introduction

Systemic lupus erythematosus (SLE) is a chronic autoimmune disease characterized by the production of multiple autoantibodies. The produced autoantibodies bind to autoantigens as immune complexes, circulating in the body, depositing in various tissues, and causing chronic inflammation (1). Failure of self-tolerance is considered the main pathogenesis of SLE, causing dysregulation in both the innate and adaptive immune systems. The upstream regulatory mechanisms that determine immune dysfunctions have been extensively documented. Based on genome-wide association studies, many scholars have attributed the pathogenesis of lupus to genetic susceptibility. Through the study of familial SLE, researchers have identified multiple loci of SLE susceptibility (2). However, the consistency of monozygotic twins with lupus is only 24–57% (3). Meanwhile, epidemiological studies of environmental exposure have shown that drugs and ultraviolet light can trigger lupus-like disease (4), suggesting that both environmental factors and genetic predisposition contribute to the development of SLE. For decades, an increasing number of studies have revealed that environmental factors play regulatory roles through epigenetic mechanisms contributing to the development of SLE, indicating that epigenetic regulation is an important contributing factor in SLE (5). This review aims to present recent advances in epigenetic factors involved in the pathogenesis, biomarkers, and therapeutic targets of SLE to facilitate an understanding of the effects of epigenetic abnormalities in SLE pathogenesis.

## 2 Epigenetic alterations in SLE

Epigenetic changes, including DNA methylation, histone modifications, noncoding RNAs (ncRNAs), and RNA methylation, are thought to be key signaling mediators between the genome and the environment. Richardson et al. reported that CD4^+^ T cells were observed to increase self-reactivity when they were treated with a DNA methylation inhibitor, 5-azacytidine (6). Since then, a series of studies have identified the function of DNA demethylation in SLE.

### 2.1 DNA methylation in SLE

**Figure 1:**
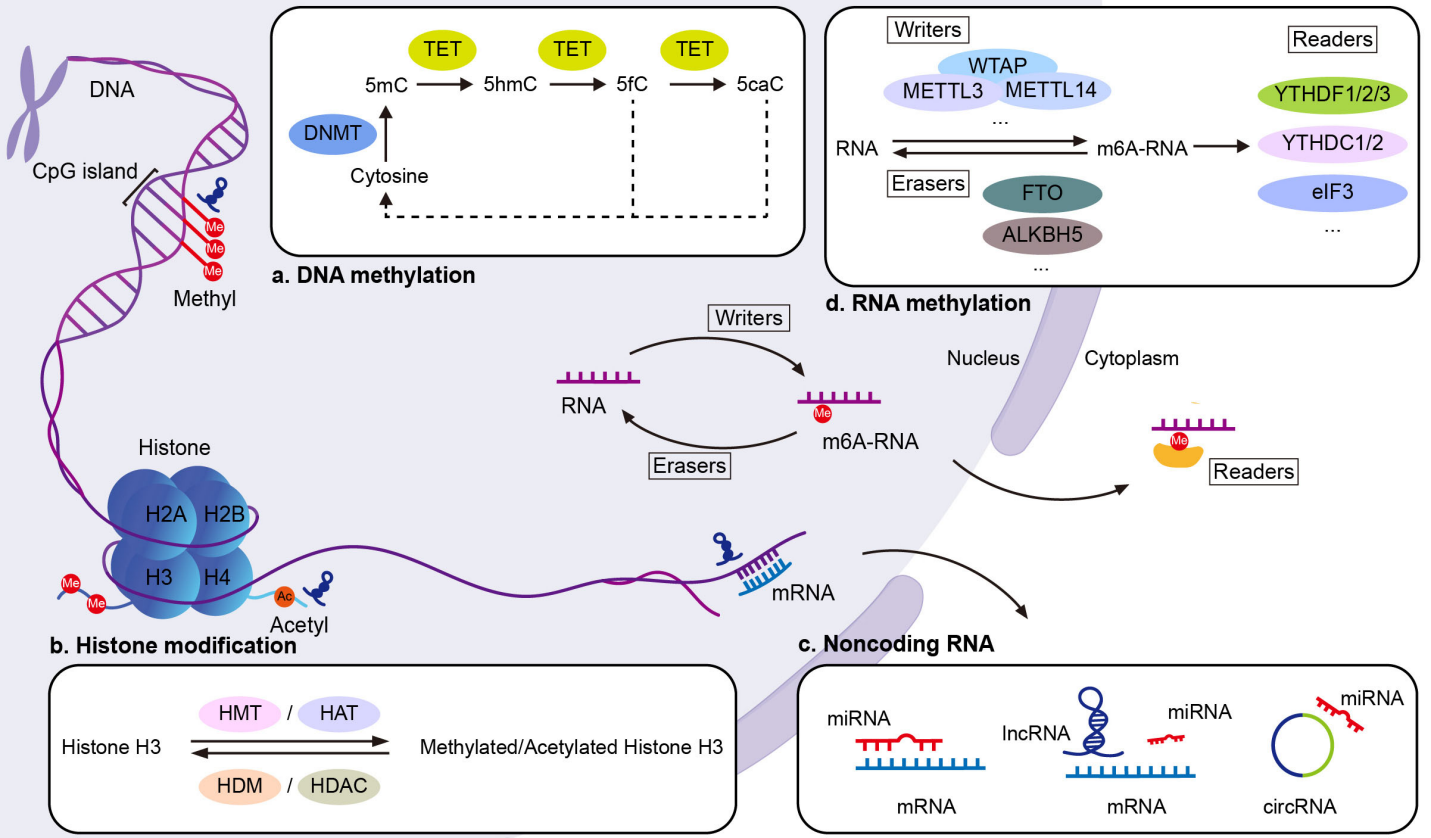
Epigenetic mechanisms in SLE. **(A)** DNA methylation: DNMTs add methyl groups to the C5 position of cytosine in CpG dinucleotides, forming 5mC. This modification silences genes by interfering with transcription factor binding. TET enzymes reverse this by converting 5mC to 5hmC, the first step in the DNA demethylation process. **(B)** Histone modifications: Modifications such as acetylation, methylation, phosphorylation, and ubiquitination target specific amino acids in histone tails, altering chromatin structure and gene expression. Histone acetyltransferases (HATs) add acetyl groups to lysine residues, activating transcription, while HDACs remove acetyl groups, repressing transcription. Histone methyltransferases (HMTs) and demethylases (HDMs) respectively add or remove methyl groups on lysine and arginine residues. **(C)** Noncoding RNAs: MiRNAs regulate gene expression by binding to complementary sequences in the 3′ untranslated region (UTR) of target mRNAs, promoting degradation or inhibiting translation. LncRNAs regulate transcription by interacting with proteins such as transcription factors, and influence translation by binding to mRNA. Both lncRNAs and circRNAs act as miRNA sponges, influencing transcriptional and post-transcriptional gene regulation. **(D)** RNA methylation: RNA methylation is regulated by three groups of enzymes: “writers” (methyltransferases), “erasers” (demethylases), and “readers” (m6A-binding proteins). This process modifies RNA function post-transcriptionally. Ac, acetyl group; eIF3, eukaryotic initiation factor 3; Me, methyl group; m6RNA, N6-methyladenosine.

DNA methylation is a dynamic process that involves both methylation and demethylation events (7). Methylation acts as a transcriptional repressive modification, which is defined as the addition of a methyl group to the C5 position of cytosine in CpG dinucleotides by DNA methyltransferase (DNMTs). The methyl groups interfere with the binding of transcription factors to DNA, thereby partially causing the silencing of those genes. Abnormal methylation patterns lead to aberrant gene activation, which contributes to SLE development. 5-Hydroxymethylcytosine (5-hmC) regulates gene transcription, resulting in dysregulation of the immune system in SLE (8). The modification of 5-hmC was found for the first time in the DNA of bacteriophages (9). Hydroxymethylation of 5-methylcytosine (5-mC) produces 5-hmC, which is further oxidized to 5-formylcytosine (5-fC) and 5-carboxycytosine (5-caC) (10). The stepwise oxidation of 5-mC is a demethylation mechanism which activates gene transcription, promotes gene expression and is catalyzed by ten-eleven translocation (TET), a methylcytosine dioxygenase (11) (**Figure 1**).

Many signaling pathways, transcription factors, and ncRNAs have been proven to affect DNA methylation patterns in CD4^+^ T cells, B cells, monocytes, neutrophils, and dendritic cells as well. DNA hypomethylation plays an important role in the pathogenesis of SLE. Studies have revealed that the hypomethylation of global genomic DNA and many immune-related genes in SLE CD4^+^ T cells result in the overexpression of growth arrest and DNA damage inducible 45 alpha (Gadd45a), CD70, CD11a, CD40L, and perforin, thereby contributing to autoimmunity (12–17). Zhao et al. found that 5-hmC was increased in SLE CD4^+^ T cells, which indicated that DNA hydroxymethylation was involved in the aberrant regulation of gene transcription in SLE pathogenesis (8). The following are the applications of DNA methylation in SLE reported over the past 2 years. More classical pathogenic mechanisms of DNA methylation in SLE over the past 5 years are listed in **Table 1**.

**Table 1:** Altered DNA methylation genes in SLE (over the past 5 years).

Methylation status	Gene	Cell type	Effects in SLE	References
DNA hypomethylation	MMP9	PBMCs; CD4^+^ T cells	Negatively correlated with creatinine and anti-dsDNA concentration and positively correlated with C3 and C4 levels. As a biomarker in the diagnosis of SLE, the diagnostic efficiency of MMP9 promoter methylation level for SLE was 0.839; Destructed autoantigens captured in immune complexes, supplemented the complement system in immune complexes clearance.	(25, 29)
	cg16797344	CD4^+^ T cells	The diagnostic efficiency of cg16797344 methylation level for LN reached around 0.8.	(27)
	S100A8	PBMCs	Undetermined.	(20)
	IFI44L	Monocytes; rNAV	Upregulating co-stimulatory receptors, inducing Th1/Th17-related cytokines, and enhancing maturation and function of Mo-DCs; Undetermined.	(21, 22)
	BCL6	Tfh cells	Increasing BCL6 expression and accelerating differentiation of Tfh cells.	(24)
	IL-17	CD4^+^ T cells	Inducing inflammatory cytokines and chemokines producing. Recruiting inflammatory cells to inflammatory organs, like monocyte and neutrophil.	(30)
	MDA5	CD4^+^ T cells	Undetermined.	(31)
DNA hypermethylation	RUNX3	PBMCs	Positively correlated with creatinine and C4 level. As a biomarker in the diagnosis of SLE, the diagnostic efficiency of RUNX3 promoter methylation level for SLE was 0.769.	(26)
	cg08332381; cg03297029	CD4^+^ T cells	As biomarkers in the diagnosis of LN, the diagnostic efficiency of cg08332381 and cg03297029 methylation level for LN reached around 0.8.	(27)
	TWEAK	Peripheral blood	Undetermined.	(18)
	CD45	PBMCs	Participating in the regulation of the expression of CD45 isoforms.	(19)
	NCR3	PBMCs	Undetermined.	(20)
	FoxP3	TFR cells	Leading to transcriptional suppression and functional decline of FoxP3.	(23)
	Global hypermethylation	Dendritic cells	Characteristic for severe LN.	(32)

Liao et al. found that the decreased mRNA expression and serum concentration of TNF-like weak inducer of apoptosis (TWEAK) significantly correlated with SLE Disease Activity Index (SLEDAI) and renal damage in SLE patients. Increased DNA methylation levels of TWEAK in the peripheral blood of SLE patients suggested that abnormal DNA methylation may participate in SLE pathogenesis by downregulating the expression of TWEAK (18). Local DNA methylation of the CD45 gene was considered to participate in the regulation of CD45 isoform expression in SLE peripheral blood mononuclear cells (PBMCs) (19). Gao reported that in identifying feature autophagy-related genes (ARGs) in SLE PBMCs, the cg24898863 (S100A8) gene was hypomethylated and upregulated, whereas the cg27490128 (NCR3) gene was hypermethylated and downregulated, prompting the possible mechanism of ARGs involved in the process of SLE (20).

Luo et al. found that the signal transducer and activator of transcription 3 (STAT3) interacted with TET2, which induced DNA demethylation of the interferon-inducible 44 like (IFI44L) promoter. Overexpression of IFI44L in monocytes can upregulate co-stimulatory receptors, induce Th1/Th17-related cytokines, and promote maturation of monocyte-derived dendritic cells (21), thus leading to autoimmunity in SLE. Hurtado et al. also reported hypomethylation of the IFI44L gene in resting naive B cells of SLE patients, suggesting that epigenetic alterations are established very early in B-cell ontogeny (22). It has been shown that the conserved noncoding sequence 2 region of forkhead box protein 3 (FoxP3) was hypermethylation in SLE follicular regulatory T cells, leading to transcriptional suppression and functional decline of FoxP3 (23). Liu et al. found that downregulation of ubiquitin-like with PHD and RING finger domains 1 (UHRF1) in the T follicular helper (Tfh) cells of SLE patients, decreased UHRF1 can reduce DNA methylation and H3K27me3 levels in the B cell lymphoma 6 (BCL6) promoter region, which resulted in the increased level of BCL6 and accelerated differentiation of Tfh cells (24). The result revealed the role of UHRF1 in regulating Tfh cell differentiation and provided a potential therapeutic target for SLE.

Recent studies have shown that the matrix metallopeptidase 9 (MMP9) promoter methylation level is significantly reduced while the runt-related transcription factor 3 (RUNX3) promoter methylation level is significantly heightened in SLE PBMCs compared to healthy controls. According to receiver operating characteristic (ROC) analysis, the diagnostic efficiency of the MMP9 promoter methylation level for SLE was 0.839 while the RUNX3 promoter methylation level for SLE was 0.769, emphasizing the potential utility of MMP9 and RUNX3 methylation levels as biomarkers for SLE diagnosis (25, 26). In the lupus nephritis (LN) group compared to both the SLE group without kidney injury and healthy controls, cg08332381 and cg03297029 were significantly hypermethylated while cg16797344 was significantly hypomethylated. According to ROC curve analysis, the diagnostic efficiency of these sites for LN was approximately 0.8, and the combined efficiency of all three sites exceeded 0.9, which emphasizes the potential use of cg08332381, cg03297029, and cg16797344 methylation levels as biomarkers for LN diagnosis (27).

Methyl-CpG binding protein 2 (MeCP2) selectively binds to 5-mC residues in CpG dinucleotides to regulate gene expression. Li et al. recently demonstrated that the overexpression of MeCP2 is markedly linked to an elevation in brain-derived neurotrophic factor (BDNF), potentially leading to disturbances in normal neuronal function in mice. C57BL/6 transgenic mice with human MeCP2 (B6.*Mecp2^Tg1^*) exhibit lupus-like phenotypes and significant central nervous system (CNS) dysfunction, making them a potential model for neuropsychiatric lupus (NPSLE) (28).

### 2.2 Histone modifications in SLE

Chromatin is composed of nucleosomes, histone octamers, and the surrounding DNA. Various modifications, such as acetylation, phosphorylation, ubiquitination, and methylation, target specific amino acids in histone tails (33), affecting the structure of chromatin and thus regulating post-translational gene expression. H3 lysine 9 trimethylation (H3K9me3) results in transcriptional inhibition (34), whereas H3 lysine 4 trimethylation (H3K4me3) is associated with transcriptional activation (35) (**Figure 1**).

In comparison with healthy controls, B cells have been observed in global histone 3 (H3) and histone 4 (H4) hypoacetylation in SLE patients (36). Zhang et al. found that in the miR-1246 promoter region of SLE B cells, H3 lysine 27 trimethylation (H3K27me3) was increased, histone H3 acetylation at Lys9 and Lys14 (H3K9/K14ac) was reduced, and downregulated miR-1246 expression led to B-cell hyperactivity (37).

Luo et al. reported that in CD4^+^ T cells of SLE, reduced suppressor of variation 3–9 homolog 1 (SUV39H1) in the cAMP-responsive element modulator α (CREMα) promoter region resulted in decreased H3K9me3 levels. The SET domain containing 1 (Set1) expression in the CREMα promoter region was increased. These resulted in decreased DNMT3a and DNA methylation levels and increased H3K4me3 levels, which promoted CREMα transcription and ultimately SLE (38). Studies have found that the reduction of jumonji domain-containing 3 (JMJD3) at the hematopoietic progenitor kinase 1 (HPK1) promoter increased H3K27me3, leading to the reduced mixed-lineage leukemia 1 and H3K4me3 abundance in Tfh cells of SLE patients. All of these resulted in HPK1 low expression and Tfh cell overactivation, ultimately inducing the development of SLE (39). Liu et al. reported that elevated M2-like phenotype in SLE monocytes was regulated by elevated acetylation levels of H3 in proliferator-activated receptor-γ (PPAR-γ) promoter. Due to the immunosuppressive function of M2-like monocytes, this study may propose a potential treatment for SLE patients (40).

There is limited research on the impact of histone modifications on the pathogenesis of NPSLE. Recent studies have found that the overexpression of MeCP2 is significantly associated with the upregulation of nuclear receptor corepressor 1 (NCoR1) and histone deacetylases (HDACs). This transcriptional dysregulation may further contribute to the development of lupus-like phenotypes in mice, along with significant central nervous system (CNS) dysfunction (28).

In this study, we present aberrant histone modifications in immune cells from SLE patients during the past 2 years. More classic studies over the past 5 years are listed in **Table 2**.

**Table 2:** Altered histone modifications in SLE (over the past 5 years).

Cell type	Gene	Alteration	Effects in SLE patients	References
B cells		Global H3 and H4 hypoacetylation	Undetermined.	(36)
	miR-1246 promoter	Increased H3K27me3 and decreased H3K9/14ac	Decreasing miR-1246 expression, leading to SLE B cell hyperactivity.	(37)
	intracellular ubiquitin-editing protein A20 promoter	Decreased H3K4me3	Downregulating A20 and promoting the proliferation of SLE B cells.	(41)
CD4^+^ T cells	CREMα promoter	Decreased H3K9me3 and increased H3K4me3	Inhibiting DNMT3a production and DNA methylation levels, promoting CREMα transcription and IL-17A production, and downregulating IL-2.	(38)
	miR-142 promoter	Increased H3K27me3 and decreased H3K9/14ac	Regulated by increased BCL-6, upregulating CD4^+^ T cells activity.	(42)
Tfh cells	HPK1 promoter	Increase H3K27me3 and decreased H3K4me3	Decreasing HPK1 expression and overactivation of Tfh cells.	(39)
	BCL6 promoter	Decreased H3K27me3	Increasing BCL6 level and accelerating differentiation of Tfh cells.	(24)
Th17 cells	STAT3; RORγT (retinoid-related orphan receptor γ T)	Phosphorylation in STAT3; Decreased H3K27me3 and increased H3K4me3 in RORγT	Activated by IL-23, causing Th17 cell maturation.	(43)
Monocytes	PPAR-γ promoter	Increased H3 acetylation	Inducing monocytes to differentiate into M2-like phenotypes.	(40)

### 2.3 Noncoding RNAs in SLE

The human genome is extensively transcribed, and over 80% of RNA transcripts are ncRNAs, which do not translate into proteins (44). NcRNAs are the most recently discovered epigenetic mechanisms (**Figure 1**). MicroRNAs (miRNAs), short noncoding RNAs, are single-stranded RNAs of 18–22 nucleotides emerging as key posttranscriptional regulators of target genes by degrading mRNAs and repressing their translation (45). Long noncoding RNAs (lncRNAs) have a length of over 200 nucleotides (46). Circular RNA (circRNA) is a type of single-stranded RNA formed as a covalently closed continuous loop. Identifying useful biomarkers for early diagnosis and treatment of SLE is a challenge in current research.

#### 2.3.1 MicroRNAs in SLE

MiRNA binds to target genes at the complementary loci in the 3′ untranslated region to regulate expression by degrading mRNA and inhibiting translation (47). MiRNA regulation plays a pivotal role in many biological processes and diseases, such as cancer and autoimmune diseases, including SLE (48).

Luo et al. found that the expression of miR-301a-3p is significantly increased in SLE PBMCs, promoting the expression of IL-6, IL-17, and interferon-γ (INF-γ), as well as IL-1 receptor-associated kinase 1 (IRAK1)-mediated Th17 cell differentiation by targeting Pellino 1 (Peli1) (49). Downregulated expression of miR-99a-3p can induce B-cell autophagy through its target gene eukaryotic translation initiation factor 4E binding protein 1 (EIF4EBP1)-mediated autophagy signaling pathway in SLE B cells (50). In SLE CD4^+^ T cells, downregulated miR-124 promotes immunoactivity by upregulating interferon regulatory factor 1 (51). In addition, upregulated miR-152-3p in SLE CD4^+^ T cells was involved in the development of SLE by targeting DNMT1 to inhibit myeloid differentiation factor 88 (MyD88) methylation and promote toll-like receptor (TLR)-mediated cellular inflammatory responses (52). In terms of innate immunity, upregulation of miR-210-5p in macrophages from SLE patients can inhibit specificity protein 1 (SP1)- and HSCARG-mediated NADPH oxidase (NOX) activity and reactive oxygen species production, leading to the accumulation of secondary necrotic cells, which is involved in the pathogenesis of SLE (53).

Chen et al. constructed a multi-miRNA detection platform based on target-triggered locked hairpin DNA-functionalized Au nanoprobes for the diagnosis and classification of SLE. The results showed that the area under curve (AUC) value for the combined signature of three urinary small extracellular vesicle (sEV) miRNAs (miR-146a, miR-29c, and miR-150) reached 1.00 (54). ElFeky et al. reported that the expression of miR-199a, miR-21, and miR-146a was significantly increased in the serum of LN patients compared to healthy controls (HCs) and SLE patients without LN. According to ROC curve analysis, the AUC values of miR-199a, miR-21, and miR-146a in distinguishing LN from SLE patients without LN were 0.96, 0.82 and 0.90, respectively. Logistic regression analysis showed that miR-199a was an independent predictor of LN with an OR of 1.69 (55).

A recent study reported that miR-155 was upregulated in regulatory T cells (Tregs) from SLE and SLE-induced mice. Inflammation-induced miR-155 impaired Treg function by decreasing suppressor of cytokine signaling-1 (SOCS1) expression. In the SLE-induced mice model, miR-155 inhibition improved Treg function under inflammatory stimulation and alleviated SLE (56). Wu et al. found that the expression of miR-125b-5p was upregulated in SLE PBMCs. After treatment with umbilical cord mesenchymal stem cells (UC-MSCs) modified by miR-125b-5p in MRL/lpr mice, the serum levels of IL-4 were elevated while IL-17A levels were reduced, and inflammatory infiltration and microthrombus formation in the lungs and kidneys were decreased (57). Targeting miR-155 and miR-125b-5p offers therapeutic approaches for alleviating SLE.

The above shows the role of miRNAs in SLE during the past 2 years. More classic studies over the past 5 years are listed in **Table 3**.

**Table 3:** MicroRNA alteration in SLE (over the past 5 years).

Cell type	MicroRNA	Alteration	Effects in SLE	References
PBMCs	miR-301a-3p	Upregulation	Promoting IL-6, IL-17 and INF-γ expression and IRAK1-mediated Th17 cell differentiation by targeting Peli1.	(49)
	miR-183-5p	Upregulation	Inhibiting FOXO1 expression. Positively correlated with SLEDAI, anti-dsDNA levels. The AUC values of miR-183-5p alone and combined with miR-374b-3p were 0.703 and 0.832, respectively.	(58)
	miR-101-3p	Downregulation	Negatively regulating inflammation in SLE by MAPK1 targeting and inhibiting NF−κB pathway. Inhibiting Th17 cell differentiation by directly targeting HDAC9.	(59, 60)
	miR-548m	Downregulation	Negatively regulating PTEN pathway.	(61)
	miR-98	Downregulation	Negatively associated with IL-6 level and regulating STAT3 phosphorylated level via IL-6.	(62)
	miR-125b-5p	Upregulation	Reducing the inflammatory infiltration and microthrombosis of lungs and kidneys of MRL/lpr mice in UC-MSCs modified by miR-125b-5p treatment group.	(57)
B cells	miR-99a-3p	Downregulation	Inducing B-cell autophagy through its target gene EIF4EBP1-mediated autophagy signaling pathway.	(50)
	miR-29a	Downregulation	Regulating target gene CRKL, and affecting IgG antibody secretion in B cells.	(63)
	miR-152-3p	Upregulation	Inhibiting KLF5 expression and increasing the BAFF expression.	(64)
	miR-326	Upregulation	Downregulating Ets-1, and promoting plasma blast development, antibody production.	(65)
CD4^+^ T cells	miR-137	Downregulation	Promoting pyroptosis and apoptosis via stimulation of AMPK pathway.	(66)
	miR-199a-3p	Upregulation	Negatively correlated with STAM and involved in JAK-STAT signaling pathway.	(67)
	miR-124	Downregulation	Promoting the immunoactivity of CD4^+^ T cells by upregulating IRF1.	(51)
	miR-152-3p	Upregulation	Associated with facial erythema, joint pain, anti-dsDNA antibody and anti-IgG antibody. Targeting DNMT1 to inhibit MyD88 methylation and promoting TLR-mediated cellular inflammatory responses.	(52)
	miR-142; miR-155; miR-499a	Downregulation	In accordance with the higher expression of the MDM2 gene, which negatively regulates p53, limiting the arrest of the cell cycle and apoptosis.	(68)
	miR-223-3p	Downregulation	Regulating T cell circulation by targeting S1PR1 in lupus-prone mice.	(69)
	miR-21	Upregulation	Associated with low complement C3.	(70)
	miR-132-3p	Upregulation	Downregulating FOXO1. Positively correlated with SLEDAI, anti-dsDNA, anti-ribosomal P and 24-hour urinary protein levels. Negatively correlated C3 and C4 levels.	(71)
Tregs	miR-155	Upregulation	Damaging the function of Treg by decreasing SOCS1 expression. In the SLE-induced mice model, miR-155 inhibition improved Treg function under inflammatory stimulation and alleviated SLE.	(56)
Monocytes and macrophages	miR-4512	Downregulation	Promoting expressions of TLR4 and CXCL2 expressions, NETs formation, and pro-inflammatory condition.	(72)
Macrophages	miR-210-5p	Upregulation	Inhibiting SP1- and HSCARG-mediated NOX activity and ROS production, leading to the accumulation of SNECs.	(53)
Dendritic cells	miR-564	Upregulation	Promoting the differentiation and maturation of dendritic cells through negative regulation of P53 expression.	(73)
Serum	miR-30e-5p	Upregulation	Targeting a variety of innate immune signal negative regulators and enhancing immune responses.	(74)
	miR-124	Downregulation	Inhibiting renal mesangial cells growth and inflammation by targeting TNF receptor-associated factor 6 (TRAF6) in patients with active lupus nephritis.	(75)
	miR-199a; miR-21 miR-146a	Upregulation	In distinguishing LN from SLE patients without LN, the AUC values were 0.96, 0.82 and 0.90, respectively. As an independent predictor of LN an independent predictor of LN, with an OR of 1.69.	(55)
	miR-381-3P	Downregulation	The AUC value was 0.803 in the SLE and HC groups. The AUC value was 0.835 in the SLE and LN groups. The lncRNA XIST/miR-381-3P/STAT1 axis may be a potential therapeutic target for LN.	(76)
	miR-200a	Downregulation	In diagnosing SLE and LN in children, the AUC value was 0.8379 and 0.7619, respectively. Positively correlated with C3, C4 and ALB levels. Negatively correlated with SLEDAI, ESR, CRP, BUN and Scr levels.	(77)
Plasma	miR-21; miR-423	Upregulation	The AUC value of miR-21 was 0.912 in the LN and HC groups. According to multivariate ROC curve analysis, the AUC value of the miR-21, -150, and -423 was 0.93 in distinguishing LN from HCs, with 79% sensitivity and 83% specificity.	(78)
	miR-150	Downregulation		
Kidney tissue	miR-127-3p	Downregulation	Related to upregulation of JAK1 and ISGS, and overactivating IFN-I signaling pathway.	(79)
	miR-183	Downregulation	Inhibiting pro-inflammatory cytokines and factors associated with renal fibrosis in human renal glomerular endothelial cells. Attenuating LN by targeting transforming growth factor beta receptor 1 (Tgfbr1), an enhancer of the TGF-β/Smad/TLR3 pathway.	(80, 81)
	miR-152	Downregulation	Downregulating MIF-induces expression of COL1A1.	(82)
sEVs	miR-146a; miR-150	Upregulation	The AUC value was 0.964, 0.740 and 0.812 for miR-146a, miR-150 and miR-29c, respectively. The AUC value of three miRNAs as as combined signature reached 1.00 for diagnosis and classification of SLE.	(54)
	miR-29c	Downregulation		

#### 2.3.2 Long noncoding RNA in SLE

LncRNAs participate in a variety of biological processes, such as silencing transcription, associating with proteins, activating protein-coding genes, binding to mRNAs, and acting as competing endogenous RNAs (ceRNAs) (83). LncRNAs have been identified to have a pivotal part in several pathogenic disorders (84) and are involved in autoimmune responses.

Nuclear enriched abundant transcript 1 (NEAT1), the best-characterized lncRNA, is known to play a crucial part in the innate immune response (85, 86). It was found that the upregulation of NEAT1 in monocyte-derived dendritic cells of SLE patients induced the expression of IL-6 and positively correlated with SLEDAI (87). Jiang et al. reported that the increased expression of NEAT1 in SLE PBMCs was negatively correlated with Th1/Th2 balance, which participated in the pathogenesis of SLE (88). LncNEAT1 contained two transcripts, lncNEAT1_1 and lncNEAT1_2, both of which were upregulated in the peripheral blood of childhood-onset SLE (cSLE). The lncNEAT1_2 expression was positively correlated with SLEDAI, fever, renal involvement, elevated ESR, and low C3 levels. According to ROC curve analysis, the AUC value was 0.812, with 62.2% sensitivity and 92.5% specificity (89).

Xiao et al. found that the expression of lncRNA growth arrest-specific transcript 5 (GAS5) was decreased in monocytes of SLE patients and negatively correlated with SLEDAI. GAS5 may be involved in TLR4-mediated inflammatory processes by inhibiting the activation of the mitogen activated protein kinase (MAPK) pathway, thus participating in the pathogenesis of SLE (90). It was also reported that lncRNA GAS5 expression was downregulated in SLE PBMCs and may contribute to SLE by targeting phosphatase and tensin homolog (PTEN) through competitively binding to miR-21 (91).

IL21 anti-sense RNA 1 (IL21-AS1) is a lncRNA located on the antisense strand of the IL21 gene motif. Liu et al. found that IL21-AS1 expression was upregulated in CD4^+^ T cells and Tfh cells from SLE patients and positively correlated with SLEDAI. Moreover, the increased acetylation levels of histone H3 on the IL21 promoter led to transcriptional activation of IL21 (92). However, a previous study reported that IL21-AS1 expression was downregulated in SLE CD4^+^ T cells and negatively correlated with SLEDAI, which may influence disease activity by participating in IL-2-mediated follicular regulatory T-cell activation in SLE (93). The different genetic backgrounds and the limited sample size in the previous study may explain the contrasting results of the two studies.

In both the serum and PBMCs of SLE patients, the expression of lncRNA H19 was increased, while that of miR-19b was decreased. Furthermore, according to ROC curve analysis, the AUC value of serum H19 was 0.853 for SLE diagnosis. Upregulation of H19 promoted apoptosis and the inflammatory response of PBMCs by interacting with miR-19b, which may participate in the pathogenesis of SLE (94). A recent study constructed a ceRNA network combined with clinical validation to screen for potential molecular markers of SLE through bioinformatics analysis. The results found that lncRNA X inactive specific transcript (XIST) and signal transducer and activator of transcription 1 (STAT1) were upregulated, while miR-381-3p was downregulated, in the peripheral blood of SLE. ROC curve analysis suggested that the lncRNA XIST/miR-381-3P/STAT1 axis could serve as a molecular marker for SLE diagnosis and a potential therapeutic target for LN (76). Liu et al. reported that lncRNA highly accelerated region 1 A (HAR1A) was significantly upregulated in PBMCs from LN patients and that it bound to miR-149-3p to upregulate SWItch/sucrose non-fermentable-related matrix-associated actin-dependent regulator of chromatin subfamily D member 1 (SMARCD1). The HAR1A/miR-149-3p/SMARCD1 pathway upregulated the expression of inducible nitric oxide synthase (iNOS), an inflammation inducer. Additionally, their study found that IL-10 secreted by iTreg cells alleviated LN through downregulating lncRNA HAR1A transcription, thereby suppressing SMARCD1-mediated iNOS activation, which might contribute to the identification of new targets for iTreg-based treatment in LN (95).

The above shows the role of lncRNA in SLE during the past 2 years. More information on studies over the past 5 years is provided in **Table 4**.

**Table 4:** LncRNA alteration in SLE (over the past 5 years).

LncRNA	Cell type	Alteration	Effects in SLE	References
NEAT1	Monocytederived dendritic cells; Myeloid-derived suppressor cells; CD4^+^ T cells; PBMCs	Upregulation	Binding to miR-365a-3p and increasing IL-6 level in moDCs. Enhancing the promotion of G-MDSCs on IFN-I signaling activation of B cells by secreting BAFF. Promoting STAT6 expression by inhibiting STAT6 ubiquitination and increasing the levels of Th2-related cytokines IL-4, IL-5 and IL-13. Negatively correlated with Th1/Th2 balance.	(87, 88, 96, 97)
LncNEAT1_2	Peripheral blood	Upregulation	Positive correlated with SLEDAI, fever, LN, elevated ESR and low C3 levels in cSLE. The AUC value was 0.812 with 62.2% sensitivity and 92.5% specificity in the HC and cSLE groups.	(89)
GAS5	Monocytes; PBMCs; CD4^+^ T cells; Plasma	Downregulation	Involved in TLR4-mediated inflammatory processes by inhibiting the activation of the MAPK pathway. Targeting PTEN through competitively binding to miR-21. Suppressing CD4^+^ T cell activation by upregulating E4BP4 via inhibiting miR-92a-3p. Acting as ceRNAs.	(90, 91, 98, 99)
IL21-AS1	CD4^+^ T cells and Tfh cells	Upregulation	Increasing histone H3 acetylation level on IL21 promoter leading to transcriptional activation of IL21 and differentiation of Tfh cells.	(92)
	CD4^+^ T cells	Downregulation	Participating in IL-2-mediated TFR cell activation.	(93)
HAR1A	PBMCs	Upregulation	Binding with miR-149-3p to upregulate SMARCD1, and further upregulated iNOS, which contributed to the pathogenesis of LN.	(95)
lncRNA XIST	PBMCs	Upregulation	The AUC value was 0.842 in the HC and SLE groups. The AUC value was 0.841 in the LN and SLE groups. The lncRNA XIST/miR-381-3P/STAT1 axis may be a potential therapeutic target for LN.	(76)
lncRNA NRIR (negative regulator of interferon response)	Serum	Upregulation	The AUC value was 0.887, with 83.6% sensitivity and 92.7% specificity. Positively correlated with SLEDAI, ESR and anti-dsDNA levels. Negatively correlated with C3 levels.	(100)
AC007278.3; HOTAIR (HOX transcript antisense intergenic RNA)	PBMCs	Upregulation	The AUC values of AC007278.3 alone and combined with HOTAIR, were 0.89 and 0.86, respectively.	(101)
ENST00000597482	PBMCs	Downregulation	Negatively correlated with the SLEDAI-2K, titres of ANA, anti-dsDNA and C4 levels. The AUC value was 0.8207 with 98.61% sensitivity and 62.22% specificity.	(102)
LINC00667; DANCR (differentiation antagonizing nonprotein coding RNA)	Plasma-derived exosomes	Upregulation	Positively correlated with the SLEDAI-2K. The AUC values were 0.815 and 0.759, respectively.	(103)
lncRNA SNHG1 (small nucleolar RNA host gene 1)	PBMCs	Upregulation	Regulating PBMCs apoptosis. Positively correlated with SLEDAI, IgG, CRP, and ESR levels. Negatively correlated with C3 and C4 levels. The AUC value was 0.899 with 81.4% sensitivity and 82.2% specificity.	(104)
RP11-273G15.2	B cells	Upregulation	Positively correlated with IFN scores and disease activity. The AUC value was 0.7557.	(105)
MIR31HG (MIR31 host gene); NKILA (NF-kappaB interacting lncRNA); PACER (p50-associated cyclooxygenase-2 extragenic RNA)	Peripheral blood	Upregulation	In the HC and SLE groups, the AUC values were 0.924, 0.954 and 0.981, respectively. In the LN and SLE groups, the AUC values were 0.893, 0.867 and 0.682, respectively.	(106)
NR_103776.1	PBMCs	Downregulation	Negatively correlated with CRP and ESR levels. The AUC value was 0.752 in the HC and SLE groups.	(107)
NONHSAT101022.2	PBMCs	Downregulation	Cis-regulating LMBRD2, inducing IFN-γ production by NK cells and enhancing β2-AR pathway.	(108)
AC007278.2	PBMCs	Upregulation	Inhibiting CCR7 transcription and promoting Tfh cell differentiation.	(109)
lncRNA H19	Serum and bone marrow-derived mesenchymal stem cells; Serum; PBMCs	Upregulation	Inhibiting BMMSCs-mediated Treg cell proliferation and differentiation by suppressing IL-2 transcription; The AUC value of serum H19 was 0.853 for SLE diagnosis. Promoting apoptosis and inflammatory response of PBMCs by interacting with miR-19b.	(94, 110)
MIAT (myocardial infarction-associated transcript)	Serum	Upregulation	In the study on MRL/lpr mice, MIAT acted as a competitive inhibitor of miR-222 to upregulate CFHR5 expression by degrading miR-222.	(111)
MALAT1 (metastasis-associated lung adenocarcinoma transcript 1)	PBMCs	Upregulation	Participating in type I IFNs-mediated SLE by upregulating OAS2, OAS3, and OASL.	(112)
TUG1	PBMCs	Downregulation	Obviously downregulated in SLE patients with LN. Positively associated with C3 levels. Negative associated with SLEDAI, ESR and 24-hour urinary protein levels. The AUC values for SLE and SLE patients with LN were 0.982 and 0.930, respectively.	(113)
RP11-2B6.2	Kidney	Upregulation	Activating the IFN-I signalling pathway by inhibiting SOCS1 expression and promoting phosphorylation of JAK1, TYK2 and STAT1.	(114)
lnc00176	CD4^+^ T cells	Upregulation	Promoting the proliferation and adhesion of CD4^+^ T cells through down-regulation of WIF1 and activation of WNT5a pathway.	(115)
lnc00892	CD4^+^ T cells	Upregulation	Targeting hnRNP K and promoting CD40L expression to activate CD4^+^ T cells and B cells	(116)
lnc0640; lnc5150	Plasma	Upregulation	The contribution may according to the MAPK signaling pathway. The AUC value of the panel of five lncRNAs (GAS5, lnc7074, linc0597, lnc0640, and lnc5150) was 0.966.	(99)
lnc7074	Plasma	Downregulation	Acting as ceRNAs.	(99)
lnc00513	Renal tissues	Upregulation	As an innovative strong regulator of type I IFN pathway.	(117)

#### 2.3.3 Circular RNAs in SLE

CircRNAs, which form covalently closed RNA circles, regulate gene expression at both transcriptional and posttranscriptional levels. Acting as miRNA sponges, circRNA can competitively bind mRNA, thereby weakening miRNA-mediated gene suppression (118, 119). Compared with miRNA and lncRNA, circRNA is more stable in mammalian cells, suggesting that circRNAs may be ideal biomarkers for human diseases. The role of circRNAs in SLE has garnered much attention in recent years.

Recently, researchers have used co-expression network analysis, bioinformatics analysis, and multilayer integrative analysis to profile the expression of circRNAs in SLE patients. Circ-calmodulin binding transcription activator 1 (CAMTA1) was found significantly decreased in SLE T cells, and was associated with disease activity. In SLE T cells, upregulated IFN-α inhibited circ-CAMTA1 expression, which may influence glucose metabolism and lead to overexpression of miR-181c-5p, thus decreased the secretion of IL-2 (120). The expression of circ-Rac GTPase activating protein 1 (RACGAP1) was downregulated in SLE PBMCs and related to SLEDAI, anti-dsDNA, and C3 levels, which participated in SLE pathogenesis by regulating the PTEN/AKT signaling pathway through binding to miR-22-3p (121). CircGARS (hsa_circ_0009000) was significantly upregulated in SLE PBMCs, which directly combined with miR-19a to regulate the expression of YTH domain-containing family protein 2 (YTHDF2) and promoted the development of SLE via the A20/NF-κB axis (122). It was also reported that increased expression of hsa_circ_0010957 in SLE CD4^+^ T cells promoted the secretion of IL-18, IL-6, and IL-17 by mediating the miR−125b/STAT3 signaling pathway, contributing to the pathogenesis of SLE (123).

Additionally, Zheng et al. explored the regulatory mechanisms of circRNAs in SLE patients, revealing a potential relationship between the circRNA–microRNA–mRNA regulatory network and pathogenesis of SLE. They dentified that 131 upregulated and 314 downregulated circRNAs in the plasma of SLE patients, with 28 upregulated and 119 downregulated circRNAs overlapping between PBMCs and plasma, which were enriched in ubiquitination, the TNF signaling pathway and the MAPK pathway. Furthermore, they constructed a network including 54 circRNAs, 41 miRNAs, and 2602 mRNAs to understand the regulatory role of circRNAs in SLE pathogenesis, suggesting that circRNAs in this network could serve as a potential diagnostic biomarker of SLE (124). Zou et al. found that circ-ETS Proto-Oncogene 1 (ETS1) was significantly downregulated in SLE CD4^+^ T cells, and positively correlated to ANA and anti-dsDNA levels while negatively correlated to C3 levels. After transfection of circETS1 overexpression, CD4^+^T cells differentiated into Treg cells, causing an imbalance in the Th17/Treg ratio. Transfection of miR-1205 mimic and si-FoxP3 reversed the effects of circETS1 overexpression. In addition, inhibition of miR-1205 had therapeutic effects in SLE mice models. Downregulation of circETS1 promoted SLE activity and inhibited Treg cell differentiation through miR-1205/FoxP3 molecular axis, which may be a novel target for SLE treatment (125).

More information on studies on circRNAs in SLE patients over the past 5 years is provided in **Table 5**.

**Table 5:** CircRNA alteration in SLE (over the past 5 years).

CircRNA	Cell type	Alteration	Effects in SLE	References
circ-CAMTA1	T cells	Downregulation	Inhibited by IFN-α, which may lead to decreased IL-2 secretion.	(120)
circETS1	CD4^+^ T cells	Downregulation	Promoting SLE activity and inhibiting Treg cell differentiation through miR-1205/FoxP3 molecular axis. Positively correlated to ANA and anti-dsDNA levels. Negatively correlated to C3 levels.	(125)
hsa_circ_0000479	Neutrophils	Upregulation	Associated with several clinical manifestations, including Raynaud’s phenomenon, alopecia and leucopenia. Positively correlated with ANA and anti-dsDNA levels. Negatively associated with absolute neutrophil count and C3.	(126)
hsa_circ_002453	Plasma	Upregulation	Correlated with the severity of kidney disorders in LN. In discriminating LN patients from controls, The AUC value was 0.906, with 90.0% sensitivity and 84.1% specificity.	(127)
hsa_circ_0001947	Plasma	Downregulation	Correlated with treatment.	(128)
hsa_circ_0044235; hsa_circ_0068367	Plasma; PBMCs	Downregulation	Hsa_circ_0044235 was related to platelet count, platelet-crit, and platelet distribution width in SLE plasma. Increasing hsa−miRNA−892a in SLE PBMCs. The AUC values of hsa_circ_0044235, hsa_circ_0068367 and the combination in SLE PBMCs were 0.873, 0.768 and 0.876, respectively.	(128, 129)
hsa_circ_0082688; hsa_circ_0082689; hsa_circ_0008675	Peripheral blood	Upregulation	All associated with C4, anti-dsDNA and anti-nucleosome levels. The AUC value of the combination of hsa_circ_0082688 and hsa_circ_0082689 was 0.823, with 91.30% sensitivity and 78.57% specificity. The AUC value of the combination of hsa_circ_0082688, hsa_circ_0082689 and anti-dsDNA was 0.987, with 95.65% sensitivity and 100.00% specificity. The AUC value of the combination of hsa_circ_0082688 and hsa_circ_0008675 was 0.925, with 79.17% sensitivity and 96.64% specificity.	(130, 131)
hsa_circ_0021372; hsa_circ_0075699; hsa_circ_0057762; hsa_circ_0003090	Peripheral blood	Upregulation	Hsa_circ_0021372 and hsa_circ_0075699 were correlated with C3 and C4. Hsa_circ_0057762 was positively associated with the SLEDAI-2K. The AUC values of hsa_circ_0057762 and hsa_circ_0003090 were 0.804 and 0.848, respectively.	(132)
circPTPN22 (protein tyrosine phosphatase non-receptor type 22)	PBMCs	Downregulation	Inhibiting proliferation and promoting apoptosis of Jurkat T cells. Acting as a miR-4689 sponge to regulate T-cell activation by targeting S1PR1. Functioning as potential disease severity indicator of SLE.	(133, 134)
circLOC101928570	PBMCs	Downregulation	Inhibiting SLE development through the miR-150-5p/c-myb/IL2RA axis.	(135)
circRACGAP1	PBMCs	Downregulation	Regulating the PTEN/AKT signalling pathway through binding to miR-22-3p.	(121)
circGARS (hsa_circ_0009000)	PBMCs	Upregulation	Combined with miR-19a to regulate YTHDF2 expression, and promoted SLE pathogenesis via the A20/NF-κB axis.	(122)
hsa_circ_0006689	PBMCs	Downregulation	Combined with anti-dsDNA and anti-Sm can increase the diagnostic sensitivity.	(136)
hsa_circ_100236; hsa_circ_102489; hsa_circ_101413	PBMCs	Upregulation	Positively correlated with SLEDAI, and associated with anti-dsDNA, thrombocytopenia, and IgG, respectively.	(137)
hsa_circ_0000479	PBMCs	Upregulation	Modulating metabolic pathways and Wnt pathway. Correlated with C3 and treatment. And combined with anti-dsDNA can increase the diagnostic efficiency.	(138, 139)
hsa_circ_0010957	CD4^+^ T cells	Upregulation	Promoting the secretion of IL-18, IL-6 and IL-17 by mediating miR−125b/STAT3 signaling pathway	(123)
hsa_circ_0012919	CD4^+^ T cells	Downregulation	Acting as miR-125a-3p sponge. Regulating the expression and methylation of MDA5. Increasing expression of DNMT1 and reversing DNA hypomethylation of CD11a and CD70, and regulated KLF13 and RANTES.	(31, 140)
hsa_circ_0123190	Renal tissues	Downregulation	Spongeing hsa-miR-483-3p, interacted with APLNR, and participated in renal fibrosis of LN.	(141)

### 2.4 RNA methylation in SLE

In recent years, methylation modifications have been reported to occur not only in DNA, but also occurs in RNA. RNA modifications are post-transcriptional and can alter RNA function. The most common RNA modification is N6-methyladenosine (m6A), which involves methylation of its adenine. M6A methylation is regulated by methyltransferases and demethyltransferases, and it is recognized by RNA binding proteins (142) (**Figure 1**). Several studies have reported that abnormal m6A modifications can contribute to the pathogenesis of autoimmune diseases by altering the expression of crucial immune factors (143, 144).

Deng et al. found that the expression of AlkB homolog 5 (ALKBH5), a demethyltransferase, was downregulated in both PBMCs and T cells of SLE patients. The expression of ALKBH5 was associated with clinical indicators in SLE, and its downregulation inhibited apoptosis and promoted T cell proliferation, potentially contributing to the development of SLE (145). Liu et al. reported the aberrant m6A methylation in SLE PBMCs. Methyltransferase 3 (METTL3) was upregulated in both SLE and the kidney of MRL/lpr mice, and it promoted the expression of interferon regulatory factor 4 (IRF4), a gene upregulated by m6A. METTL3 induced kidney damage by promoting IRF4-mediated plasma cell infiltration in an m6A-dependent manner (146). Tian et al. identified a causal relationship between MMP9, an m6A-related gene, and ischemic stroke in SLE. As a biomarker for ischemic stroke in SLE, the odds ratio (OR) was 1.0134 (147). Zhao et al. confirmed that the levels of METTL3, Wilms tumor 1 associated protein (WTAP), YTH domain containing 2 (YTHDC2), YTHDF1, fragile X mental retardation 1 (FMR1), and fat mass and obesity-related protein (FTO) in the glomeruli could effectively distinguish patients with LN from healthy individuals, and that they are correlated with the glomerular filtration rate (GFR) and activated natural killer (NK) cells, indicating that they are potential prognostic biomarkers (148).

More classical pathogenic mechanisms of RNA methylation in SLE over the past 5 years are listed in **Table 6**.

**Table 6:** Altered RNA methylation genes in SLE (over the past 5 years).

Regulators	Cell type	Alteration	Effects in SLE	References
METTL3	PBMCs	Upregulation	Inducing kidney damage through promoting IRF4-mediated plasma cell infiltration via an m6A-dependent manner.	(146)
MMP9	PBMCs	Upregulation	As a biomarker for ischemic stroke in SLE, with an OR of 1.0134.	(147)
ALKBH5	PBMCs; T cells	Downregulation	Positively related with C3 and C4 levels. Negatively related with SLEDAI, anti-dsDNA level and erythrocyte sedimentation rate. Downregulated ALKBH5 inhibited apoptosis and promoted the proliferation of T cells.	(145, 149)
METTL14 (Methyltransferase 14); YTHDF2	PBMCs	Downregulation	Associated with white blood cell count and monocyte count; Associated with C3 and fever. Decreased YTHDF2 expression was a risk factor for SLE.	(149)
METTL3; WTAP; YTHDC2; YTHDF1; FMR1; FTO	Glomeruli	Downregulation	Correlated with (GFR) and activated NK cells. Distinguish LN and healthy individuals.	(148)

## 3 Epigenetic factors as potential biomarkers and therapeutic targets for SLE

**Figure 2:**
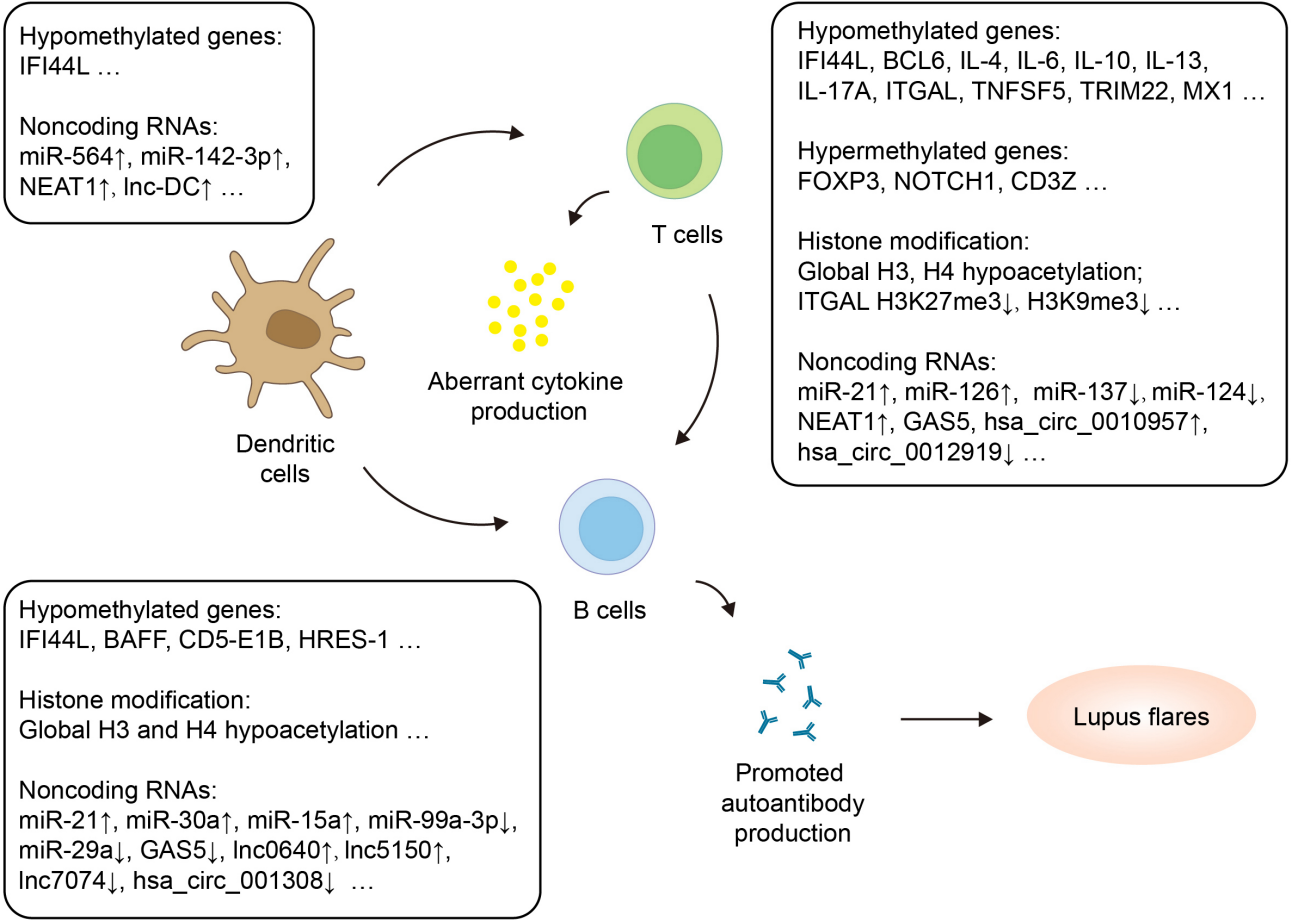
Epigenetic regulation of adaptive immune cells in SLE. Adaptive immune cells with altered epigenetic marks in SLE include dendritic cells, T cells and B cells. The figure highlights hypermethylated and hypomethylated genes, histone modification as well as dysregulated noncoding RNAs. CD5-E1B, CD5 protein and exon 1B; HRSE-1, HTLV-1-related endogenous sequence 1; ITGAL, Integrin Subunit Alpha L; MX1, Myxoma resistance 1; NOTCH1, Neurogenic locus notch homolog protein 1; TRIM22, Tripartite Motif Containing 22.

As currently available biomarkers of SLE, such as autoantibodies, have limitations in early diagnosis, it is crucial to explore new biomarkers that have high specificity and sensitivity for diagnosing the disease and assessing the severity. Increasing evidence suggests that dysregulated epigenetic modifications in immune cells play a significant role in the pathogenesis of SLE (**Figure 2**). These epigenetic changes have also been identified as potential biomarkers and therapeutic targets.

DNA methylation is currently the most studied and stable epigenetic modification. The methylation level of the IFI44L promoter has been suggested as a marker that can diagnose in the early stage and potentially predict specific disease manifestations in SLE (150). Zhang et al. published a study on a high-resolution melting-quantitative polymerase chain reaction assay to detect the methylation of the IFI44L promoter for the diagnosis of SLE, which has good consistency with previous pyrosequencing and is simpler and more economical (151). MicroRNAs are smaller than the transcripts of protein-coding genes, which makes microRNAs more resistant to degradation by endogenous RNase enzymes. Moreover, miRNAs have been found in body fluids such as plasma and urine (152), which makes the test samples easier to obtain. Therefore, miRNAs are important biomarkers for the diagnosis, staging, classification, and prognosis of SLE. In addition to the pathogenesis, miRNA dysregulation is related to disease activity, autoantibody production, organ damage, and therapeutic effects. Chen et al. constructed a multi-miRNA detection platform using target-triggered locked hairpin DNA-functionalized Au nanoprobes for the diagnosis and classification of SLE. This platform achieved an AUC value of 1.00 for a combined urinary sEV miRNA signature (miR-146a, miR-29c, and miR-150). It exhibited good practicability in SLE diagnosis, offering advantages such as low cost, rapidity, high sensitivity, and noninvasiveness (54). Compared with miRNAs, lncRNAs are more tissue-specific and biologically complex (46). Therefore, lncRNA is even more advantageous than miRNA as a new biomarker. Many studies have shown that lncRNAs can be used as potential biomarkers for SLE. For instance, upregulation of H19 promoted apoptosis and the inflammatory response of PBMCs by interacting with miR-19b, contributing to the pathogenesis of SLE. And the AUC value of H19 was 0.853 for SLE diagnosis (94). Additionally, lncNEAT1_2 expression was positively correlated with disease activity in cSLE. According to ROC curve analysis, the AUC value was 0.812, with 62.2% sensitivity and 92.5% specificity, suggesting that lncNEAT1_2 may be a potential biomarker for cSLE (89). Chen et al. constructed a ceRNA network combined with clinical validation to screen for potential molecular markers of SLE through bioinformatics analysis. The results confirmed that the lncRNA XIST/miR-381-3P/STAT1 axis could serve as a molecular marker for SLE diagnosis (76). CircRNAs have become a focus of research in many human diseases. Many studies have revealed that circRNAs can manipulate miRNAs and thus have great potential for clinical applications in diseases such as SLE. There have been many studies demonstrating the circRNA expression profile in SLE through various technologies. Through these analyses, researchers have proved that many circRNAs have characteristics for the prediction of disease progression. A circRNA–microRNA–mRNA regulatory network including 54 circRNAs, 41 miRNAs, and 2602 mRNAs was constructed to understand the regulatory role of circRNAs in SLE pathogenesis, which could be potential diagnostic biomarkers of SLE (124).

Epigenetic modifications can be used not only as potential biomarkers, but also as potential therapeutic targets for lupus. In the SLE mouse model, miR-155 inhibition improved Treg function under inflammatory stimulation and alleviated SLE (56). After treatment with UC-MSCs modified by miR-125b-5p in MRL/lpr mice, and inflammatory infiltration and microthrombus formation in the lungs and kidneys were reduced (57). In addition, downregulation of circETS1 promoted SLE activity and inhibited Treg cell differentiation through miR-1205/FoxP3 molecular axis, which may be a novel target for SLE treatment (125).

More potential epigenetic biomarkers and potential therapeutic targets for SLE over the past 5 years are listed in **Tables 7** and **8**.

**Table 7:** Potential epigenetic biomarkers for SLE (over the past 5 years).

Modification	Gene	References
DNA methylation	MMP9, IFI44L, RUNX3, cg16797344, cg08332381, cg03297029	(21, 25–27)
MicroRNA	miR-301a-3p, miR-146a, miR-150, miR-29c, miR-199a, miR-21, miR-183-5p, miR-101-3p, miR-548m, miR-137, miR-199a-3p, miR-132-3p, miR-124, miR-381-3P, miR-200a, miR-423	(49, 54, 55, 58, 61, 66, 67, 70, 71, 75–78)
LncRNA	lncRNA XIST, NEAT1, lncNEAT1_2, lncRNA H19, GAS5, lncRNA NRIR, AC007278.3, HOTAIR, ENST00000597482, LINC00667, DANCR, lncRNA SNHG1, RP11-273G15.2, NKILA, PACER, NR_103776.1, NONHSAT101022.2, AC007278.2, lnc0640, lnc5150, lnc7074, TUG1	(76, 87, 89, 94, 99–109, 113)
CircRNA	circGARS, hsa_circ_0010957, hsa_circ_002453, hsa_circ_0001947, hsa_circ_0044235, hsa_circ_0068367, hsa_circ_0082688, hsa_circ_0082689, hsa_circ_0008675, hsa_circ_0057762, hsa_circ_0003090, circLOC101928570, hsa_circ_0006689, hsa_circ_100236, hsa_circ_102489, hsa_circ_101413, hsa_circ_0000479, circPTPN22, hsa_circ_0012919	(122, 123, 127–132, 134–140)
RNA methylation	MMP9, METTL3, WTAP, YTHDC2, YTHDF1, FMR1, FTO	(147, 148)

**Table 8:** Potential therapeutic targets for SLE (over the past 5 years).

Modification	Gene	References
DNA methylation	IL-17	(30)
Histone modification	STAT3, RORγT, PPAR-γ promoter	(40, 43)
MicroRNA	miR-99a-3p, miR-152-3p, miR-210-5p, miR-155, miR-125b-5p, miR-101-3p, miR-98, miR-29a, miR-137, miR-4512, miR-30e-5p, miR-381-3P, miR-183, miR-152	(50, 52, 53, 56, 57, 59, 60, 62, 63, 66, 72, 74, 76, 80–82)
LncRNA	lncRNA XIST, GAS5, IL21-AS1, HAR1A, AC007278.3, HOTAIR, AC007278.2, lncRNA H19, RP11-2B6.2, lnc00176, lnc00892	(76, 90, 92, 93, 95, 98, 101, 109, 110, 114–116)
CircRNA	circRACGAP1, circGARS, hsa_circ_0010957, circETS1, circLOC101928570	(121–123, 125, 135)

## 4 Conclusions

Discoveries of epigenetic modifications have extended our knowledge of complex regulation in genes and added new insights into understanding the pathogenesis of SLE. There are still challenges in ascertaining the mechanism of epigenetic changes in the occurrence and development of the disease. Epigenetic events have great potential in finding targets for individualized treatment interventions and disease diagnosis biomarkers. With the increasing attention to epigenetic mechanisms, more surprising discoveries will provide new ideas for the treatment of SLE.
